# Motivation to participate in secondary science communication

**DOI:** 10.3389/fpsyg.2022.961846

**Published:** 2022-09-08

**Authors:** Zhichen Hu, Baolong Ma, Rubing Bai

**Affiliations:** School of Management and Economics, Beijing Institute of Technology, Beijing, China

**Keywords:** secondary science communication, audience dissemination/participation, social media, theory of consumption values, theory of planned behavior

## Abstract

The rise of social media provides convenient mechanisms for audiences to participate in secondary science communication (SSC). The present study employs the theory of consumption values and theory of planned behavior to predict audiences’ SSC intentions. The results indicate that emotional value, social value, altruistic value, attitude, internal perceived behavioral control and subjective norm are significant predictors of audiences’ intentions to share or to repost science content on their social media. These results suggest that the theory of consumption values, together with the theory of planned behavior, is a useful framework for understanding SSC behaviors.

## Introduction

**Figure 1:**
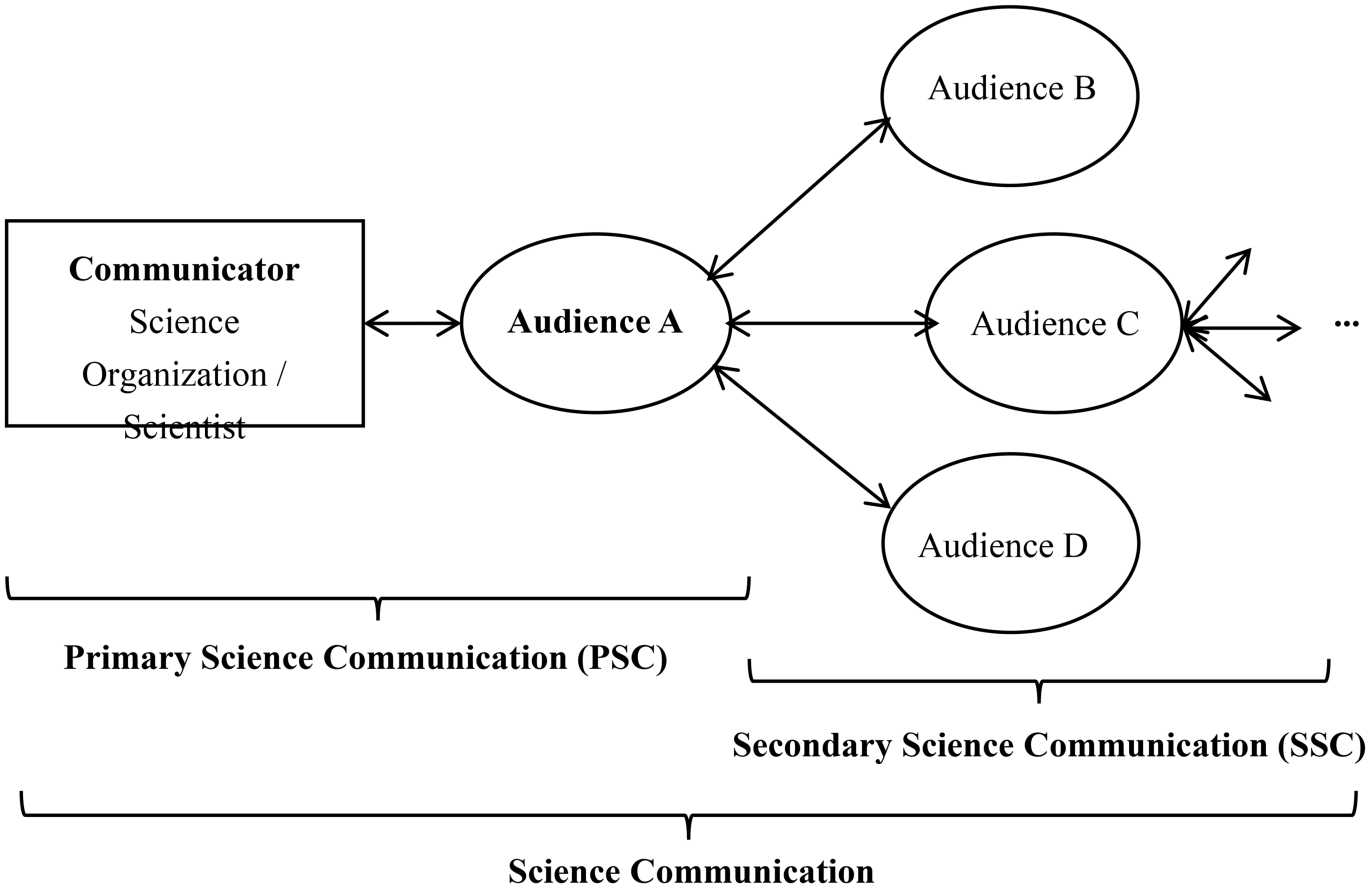
The relationship between PSC and SSC.

The rise of social media has dramatically transformed the way the audiences engage or interact with science communicators. For a long time, science organizations and scientists have been delivering knowledge to audiences through science cafes (Dallas, 2014), science festivals (Jensen and Buckley, 2014; Boyette and Ramsey, 2019; Nielsen et al., 2019), Web and e-mails (Duke, 2002), etc. With social media, science communication practitioners’ work has become increasingly fast-paced--leaving less time for investigation, storytelling and curating what information should be disseminated (Massarani et al., 2021). Compared with the traditional science communication platforms, social media is recognized as a more efficient one for science communicators and audiences due to its advantages of widespread, faster speed, deeper interactivity and visibility (Lee and VanDyke, 2015; Su et al., 2017; Lee et al., 2018). For example, institutions and scientists can share their research on Twitter, Instagram, WeChat, and Weibo in a direct and instantaneous fashion (Lee and VanDyke, 2015). And audiences can engage with these updates through a few ways, including “Like,” “Comment,” “Share” and “Repost” (Hwong et al., 2017). More importantly, social media extends the traditional role of audience, typically providing them with roles of the “audience” or the “communicator,” or both of them, according to the stage they are in, as shown in Figure 1. By evaluating and selectively disseminating the message originally posted by an organization or a scientist, audience can help circulate information to a wider group than the organization’s own pool of followers, thus facilitating new rounds of discussions and greatly enhancing science communication effectiveness (Boyd et al., 2010; Lovejoy and Saxton, 2012; Hermida et al., 2014; Su et al., 2017).

A growing body of research shows that scientists consider public communication in a relatively simple way (Davies, 2008; Jensen et al., 2008; Peters et al., 2008; Dunwoody et al., 2009; Nisbet and Markowitz, 2015; Besley et al., 2016; Choi and Kim, 2020), indicating that communicators’ interaction with their audience in the digital public sphere requires more cautiousness for fear of criticism and personal attacks, or being framed by suggestive questions and potentially receiving low-quality comments from audience. And these could in turn lead to a decline of trust in similar communicators or scientists on the platform (Dermentzi and Papagiannidis, 2018; Hara et al., 2019; Jones et al., 2019; Sajeev et al., 2019). Moreover, research has shown that scientists often approach their engagement work through a narrow set of skills and considerations (Franks et al., 2013; Peters, 2013; Grand et al., 2015; Dudo and Besley, 2016; Besley et al., 2018; Yuan et al., 2018). Meanwhile, the science communication ecosystem is seen fragmented, with numerous interfaces where professional science communicators and new science communication actors interact with audiences in myriad ways (Bubela et al., 2009; Rutsaert et al., 2013). Therefore, science organizations and scientists show limited influence on social media (McClain, 2019), which further highlights the importance of audiences acting as “communicators” and disseminating science content on social media.

However, according to the academic research and science communication literature, in fact, audiences do not always participate in the online dissemination: Audience engagement in online science content is transient and brief (Thaler et al., 2012), and institutions lack clear plans for the goals and expected outcomes of online communication (Bik et al., 2015). Most audiences only share science content in a single-connected community, but they could have spread it to new audiences or those who in great need of scientific education (Williams et al., 2015). Although the patterns of dissemination differ over time, the impact of science content on the public still remains limited (Alperin et al., 2019), and engagement of each online user tends to decline as the audience size increases (Kahle et al., 2016).

What’s worse, the lack of online gatekeepers to distinguish between correct interpretations of peer-reviewed data and personal opinions, coupled with people’s disposition to communicate in isolated communities, can lead to the spreading and reinforcement of false information, hereafter referred to as misinformation (Bessi et al., 2015; Radzikowski et al., 2016). The misinformation present on social media can spread even more quickly to a broad range of people in a short amount of time, increasing the likelihood that information seekers will notice misinformation and incorporate it into their worldviews (Del Vicario et al., 2016; Vosoughi et al., 2018). These effects are aggravated when media consumers have a low level of media literacy and are not able to recognize misinformation (Scheufele and Krause, 2019). Therefore, a clear understanding of the causes of SSC is crucial for effectively managing the science communication.

This study focuses on the predictors of secondary science communication (SSC) on social media. Specifically, this study entails a survey designed to better understand audiences’ SSC behaviors. Previous literature has defined the transmission of information among audiences as “retransmission” (Kim et al., 2013; Luarn et al., 2014; Sutton et al., 2015), but this definition only applies to the direct repetition of original content, ignoring the audience’s right to screen and evaluate or even reprocess information and the special attributes of social media context. Thus, we define “SSC” as audience’s selective dissemination of the original science information from professional communicators to other audiences according to their social scope and preference, in which an information reprocessing may be included.

Our definition clarifies the following four characteristics of SSC: (1) SSC differs from PSC. In the case of PSC (as shown in Figure 1), professional organizations or individuals post science content which is new to the network. When this science content is shared by audiences, SSC has occurred. More specifically, SSC is the reposting of science content already on the Internet. Both SSC and PSC are forms of science communication, as each process involves communication of science content to others (Dockter et al., 2020). (2) SSC also often occurs offline, but social media provides a lower threshold and a wider platform for SSC. In this study, we focus on the SSC behavior online, mostly on social media, including sharing science content with specific friends or in Chat Group, @mentions friends on Twitter, reposting it to WeChat Moments, and modified reposting, etc. (3) “SSC” concept emphasizes audience’s value-based evaluation and selective dissemination. In other words, audiences will not always participate in the SSC, but will disseminate specific content based on their own time, resources, preferences and evaluations. Compared with “retransmission,” “SSC” represents a conscious shift away from one-way, linear dissemination of scientific information to a bidirectional, participatory, deliberative communication in which the audiences are active participants (Kurath and Gisler, 2009). (4) Resharing the science content shared by an audience is also regarded as SSC, and the information may be reprocessed in this series of processes. The science content produced by professional institutions may be boring and difficult to understand, but it can be more colorful, interesting and credible after being slightly processed by the audience (Welbourne and Grant, 2016; Dockter et al., 2020).

## Literature review

Science communication plays a number of vital roles in society (Fischhoff and Scheufele, 2013; Davies, 2021), including effectively engaging with diverse stakeholders (Weingart and Joubert, 2019), combating misinformation (Goldstein et al., 2020), and encouraging wider participation in Science, Technology, Engineering and Mathematics which is known as STEM (Bevan et al., 2020). Burns et al. (2003) recognize five main purposes of science communication: awareness, enjoyment, interest, opinion formation, and understanding. Through a qualitative interview study with scholars and professors, Davies (2021) proposed six types of roles for science communication within society. It ensures the accountability and legitimacy of publicly funded science, has practical value (such as enabling laypeople and policy makers to make good choices in today’s technologically oriented societies) and enhances democracy by empowering citizens. Moreover, science communication allows access to the beauty of science as an aspect of culture, serves promotional purposes and casts economic effects in recruiting people into scientific careers or preparing a market base for technological innovations. In general, emphasizing the functions of public communication and engagement with science for democracy, science communication is defined as a process where information is transferred or negotiated, as well as a means through which societies can nurture shared sense-making about the issues that face them (Weingart and Joubert, 2019; Davies, 2021).

While science communication literature calls on communicators and audiences to interact and build relationships on social media (Taylor and Kent, 2014; Avidar et al., 2015), there is still a lack of research on the predictors of audience dissemination. Previous research related to social media focuses mostly on scientists’ motivation for choosing online platforms (Poliakoff and Webb, 2007; Mewburn and Thomson, 2013; Jia et al., 2017), how effectively science institutions are using social media (Lee and VanDyke, 2015; Lee et al., 2018), core topics around scientific issues (Pearce et al., 2014; Büchi, 2017; Lee et al., 2020), audience’s trust in online science content (Liang et al., 2014; Huber et al., 2019) as well as the extent to which audiences use social media (Anderson et al., 2010; Su et al., 2015; Hargittai et al., 2018). However, little has examined audience’s SSC behavior. Moreover, given the scarcity of systematic analysis into the mechanisms of SSC, current practices of science communication on social media are based on intuition and experiences rather than empirical evidence. To be more specific, a deeper understanding of the predictors of SSC can help communicators to better manage SSC. Specifically, institutions and scientists can adopt different communication strategies (such as providing either more functional value or altruistic value) for different science content and adjust their strategies in time according to the feedback of the audiences (such as Like, Comment, Share and Repost; Das et al., 2021; Li and Guo, 2021) and finally contributes to a more effective science communication.

Behavioral theories believe that any type of behavior, SSC included, can be predicted by psychological variables. Two theories can be particularly helpful for predicting audiences’ intentions to participate in SSC: Theory of consumption values (TCV) and Theory of planned behavior (TPB).

### Theory of consumption values

TCV was proposed by Sheth et al. (1991) to explain why consumers choose one product over the others. Consumption value is the estimate of product utility that consumers received compared to their effort to reach or consume the product, and it is also the key determinant of consumers’ attitude and behavior (Zeithaml, 1988). Marketing scholars believe that there are two types of motivation for consumer behavior: functional needs and nonfunctional needs. Based on this concept, consumption value can be segmented into specific factors, such as functional, emotional, and social value. The importance of consumption value theory lies in the assumption that consumers balance value assessments to make informed internal and external decisions, which is more than just purchasing behavior (Kim et al., 2007).

TCV frameworks has been used in a variety of domains and thus additional value dimensions have been developed within the recent years. For example, Sweeney and Soutar (2001) proposed quality, emotional, price, and social values in a retail purchase situation; Turel et al. (2010) confirmed visual appeal, social, playfulness, and value-for-money as factors that affect word-of-mouth intentions of hedonic digital artifacts; Kim and Chang (2020) applied economic, emotional, social, and altruistic values to investigate the intention to take part in festivals. And in the domain of social media, TCV has been used to study the intention to pay for social networking sites (Lu and Hsiao, 2010), usage construct with regard to Facebook (Aladwani, 2014), and reason of using online social media brand community (Kaur et al., 2018). However, to our best knowledge, the domain of science communication has not been examined from the perspective of TCV.

Science communication has been proposed on the assumption that ignorance is the basis of a lack of societal support for various issues in science and technology. This model is known as the knowledge deficit model of science communication (Simis et al., 2016). Deficit model thinking proposes the belief that public skepticism toward modern science is caused by a lack of adequate knowledge about it. Furthermore, this skepticism, or “knowledge deficit,” can be overcome by providing sufficient information to the public (Besley and Tanner, 2011). This model adopts a one-way, top-down communication process, in which scientists—with all the required information—filled the knowledge vacuum in the scientifically illiterate general public as they saw fit. Scientific facts and methods were the vital components of public understanding for the deficit model (Miller, 2001). For example, science communication studies find that audiences have deficient knowledge about science with a lack of interest and low trust in it (Durant, 1999; Bauer et al., 2007), and that providing the audiences with knowledge about science increases their motivation to process scientific information (Bauer, 2016; Hargittai et al., 2018). Therefore, science content on social media needs to possess high values and interestingness to attract audiences to engage with messages (Hwong et al., 2017).

On the other hand, online science content can be perceived as a service provided by communicators (Su et al., 2015; Bai et al., 2019). And communicators use affordances of social media to engage in presentation of science content to garner public attention to science (Bhargava and Velasquez, 2021). Attention is a key resource for social movements (Davenport and Beck, 2001). Tufekci (2013) suggested that attention is the means through which a social movement can introduce and fight for its preferred framing, convince broader publics of its cause, recruit new members, attempt to neutralize opposition framing, access solidarity, and mobilize its own adherents. Therefore, communicators need to provide audiences with content that interests them as much as possible to get more attention. For example, the science content “liked” by audiences on social media can be widely disseminated, while as for those that audiences disliked tend to have a lower number of comments and retweets, ultimately resulting in poorer communication. In other words, SSC can be partially explained by “Attention Economy” and represents audiences’ attention consumption choice-oriented behavior. Thus, TCV is appropriate for predicting audiences’ intention to participate in SSC.

In the present study, we apply functional, emotional, social, and altruistic value to explain audiences’ SSC intentions. Specifically, functional value refers to “*audience perceived utility acquired from the expected performance of the online communicator or science content*.” Emotional value stands for “*audience perceived utility acquired from the feelings or affective states that science content generates*.” Social value represents “*audience perceived utility acquired from the enhanced social self-concept by following the online communicator*.” Altruistic value denotes “*audience perceived utility acquired from helping others through gaining science knowledge*” (Sweeney and Soutar, 2001; Kim and Chang, 2020).

### Theory of planned behavior

TPB was proposed by Ajzen (1991), suggesting that an individual’s behavior is affected by the attitude toward the behavior, what other people think of the behavior, and how much control has over the expected barriers. TPB has received widespread support as a model of behavior and has been adopted to understand audience’s adoption of communicated information (Paulussen et al., 1995; Longnecker, 2016), environmental civic engagement (Ho et al., 2015; Witzling et al., 2015), educators’ attitudes toward science (van Aalderen-Smeets et al., 2012), and scientists’ communication behavior (Breslin et al., 2001; Poliakoff and Webb, 2007; Dijkstra et al., 2015).

Similarly, TPB frameworks are also useful for understanding SSC behavior. To be more specific, if an audience does not consider it important to repost science knowledge, the audience will hardly participate in SSC no matter how attractive the science content seems to be (Attitude). And with a high level of media literacy (Internal perceived behavioral control) and abundant time (External perceived behavioral control), one can confidently recognize misinformation and consider it easy to repost science content on social media, then the audience will be likely to participate in SSC as well. In addition, whether the audience’s friends frequently repost science content on social media can also influence the SSC intention (Subjective norm and objective norm). In other words, if both an audience and their peers believe SSC is important, then the audience will be more willing to participate in SSC. Williams et al. (2015) found that science content is more easily spread in a single-connected community, which supports the influence of subjective norm and objective norm from the side.

Therefore, this study applies attitude, internal perceived behavioral control, external perceived behavioral control, subjective norm, and descriptive norm to predict SSC intention. Based on the original definitions of the variables in TPB as well as the research background of this study, attitude means “*audience’s attitude toward SSC behavior*.” Internal perceived behavioral control refers to “*audience’s internal perception that he possesses control over personal resources to participate in SSC, such as confidence, adequate planning, and the ability*.” External perceived behavioral control stands for “*audience’s internal perception that he has control over external conditions and situations to participate in SSC, such as time, channels, and availability*.” Subjective norm signifies “*audience’s perception of significant referents’ opinions toward SSC behavior*.” Descriptive norm means “*audience’s perception of significant referents’ typically SSC behavior*” (Armitage et al., 1999; Armitage and Conner, 1999; Poliakoff and Webb, 2007).

### Hypotheses

This study examines the extent to which audiences’ SSC intentions are predicted by TCV-based predictors (Functional value, Emotional value, Social value, Altruistic value) as well as by TPB-based predictors (Attitude, Internal perceived behavioral control, External perceived behavioral control, Subjective norm, Descriptive norm).

Although, as far as we consider, there is no science communication research based on TCV, audience’s SSC is essentially a form of consumption choice-oriented behavior (Su et al., 2015). Thus, we argue that the higher the values of science content, the higher the audiences’ intentions to be involved in SSC. Precisely speaking, we propose that perceived functional value positively affects SSC intentions (Hypothesis 1a); perceived emotional value positively affects SSC intentions (Hypothesis 1b); perceived social value positively affects SSC intentions (Hypothesis 1c); perceived altruistic value positively affects SSC intentions (Hypothesis 1d).

In addition, Poliakoff and Webb (2007) indicates that attitude, perceived behavioral control, and descriptive norms can positively predict scientist’s intentions to participate in public engagement activities. These effects may be similar between the scientist and the audience. Therefore, we propose that attitude positively affects SSC intentions (Hypothesis 2a); internal perceived behavioral control positively affects SSC intentions (Hypothesis 2b); external perceived behavioral control positively affects SSC intentions (Hypothesis 2c); subjective norm positively affects SSC intentions (Hypothesis 2d); descriptive norm positively affects SSC intentions (Hypothesis 2e).

## Materials and methods

### Participants

We commissioned a sample service company^1^ to collect data. Sojump.com has 8.39 million panel members in China. We designated that only participants who followed (at least) a science communicator on social media could participate in the survey. The final sample consisted 489 complete cases, after deleting 74 completions that failed a series of filter questions (including 3 reverse scale items).

In terms of the participants, 63.2% were female, and their average age was 26 years (*SD* = 5.58). Participants with postgraduate degree account for 12.5% of the entire sample, undergraduate 81.0% while high school or below takes up 6.5%. Furthermore, the majority of the participants earned 5,001–10,000 yuan per month (40.3%). Altogether, 40.1% of the participants earned less than 5,000 yuan per month, 13.3% earned 10,001–15,000 yuan, 4.1% earned 15,001–20,000 yuan, and 2.2% earned more than 20,001 yuan monthly. According to the National Bureau of Statistics (2021), the annual *per capita* disposable income of China was 35,100 yuan, and the ratio of male to female population in China is around 51.2%. Therefore, the sample distribution is basically consistent with the national conditions.

### Questionnaire

According to Huang and Yang (2020); Taragin-Zeller et al. (2020); and Besley et al. (2021), at the beginning of the questionnaire, participants read: “With the development of the Internet, many science organizations or individual communicators have appeared on social media, such as Guokr, Kepuchina.cn, UFO Talk.^2^ They spread science knowledge through social media like WeChat public account, micro-blog, TikTok and so on. At the same time, people can also share and repost their science content to their friends on these social media.” The above-mentioned science communicators have all got a certification as “popular science communicator” on the social media platforms. Then the subjects were asked to recall a science communicator they had followed on social media and fill in the questionnaire according to their perceptions. Participants were required to write down the name of the communicator, their content (whether the communicator mainly spread science knowledge through articles or videos), and the frequency they went through the content posted by the communicator.

The communicators listed by the participants are shown in Table 1.

**Table 1:** Information of the communicators listed by participants.

Name	N (%)	Org/Ind	Content	Social media	Field
**Guokr.com**	47 (9.61%)	Organization	Article	WeChat	Technology
**Kepuchina.cn**	31 (6.34%)	Organization	Article	WeChat	Technology
Museum Magazine	25 (5.11%)	Organization	Video	Weibo	Nature
**UFO Talk**	22 (4.50%)	Organization	Video	Youku	Sex
XueShu	18 (3.68%)	Individual	Article	Weibo	Nature
Paper Clip	15 (3.07%)	Organization	Video	Bilibili	Common sense
ZhangChenliang	11 (2.25%)	Individual	Video	TikTok	Nature
Cas.cn	11 (2.25%)	Organization	Article	WeChat	Technology
**Songshuhui**	9 (1.84%)	Organization	Article	Weibo	Nature
**Huxiu.com**	7 (1.43%)	Organization	Article	WeChat	Business
LiYongle	7 (1.43%)	Individual	Video	TikTok	Technology
HuaXiaoluo	7 (1.43%)	Individual	Video	Weibo	Health
NASA Lovers	6 (1.23%)	Individual	Video	Weibo	Universe
BiXiaotian	6 (1.23%)	Individual	Video	Bilibili	Technology
Dr. ThreeOne	5 (1.02%)	Individual	Video	Toutiao	Technology
Science Traveler	5 (1.02%)	Individual	Video	Weibo	Nature
Approaching Science	5 (1.02%)	Organization	Video	CCTV	Nature
Dxy.cn	5 (1.02%)	Organization	Article	Weibo	Medicine
Others	247 (50.5%)	—	—	—	—

N = 489. The Names in bold are the communicators that we presented as the examples to the participants.

Items adopted in this research were all taken from their English versions and translated and them to the Chinese setting. Two different bilingual researchers translated the English versions of the scales into Chinese and translated them back into English to make sure the meanings of the items remain the same in Chinses and English. All the seven-point (1 = *strongly disagree*, 7 = *strongly agree*) measurements of variables are borrowed from previous studies. The questionnaire ends with demographic questions, including gender, age, education and monthly income.

*Functional value* was measured with three items (Sweeney and Soutar, 2001): “I think the content produced by this communicator is well made,” “I think the content produced by this communicator meets my needs,” “I think this communicator has provided me with good help” (Alpha coefficient = 0.83).

*Emotional value* was measured with three items (Sweeney and Soutar, 2001): “The content produced by this communicator will make me feel good,” “The content produced by this communicator is something that I would enjoy,” “The content produced by this communicator would give me pleasure” (Alpha coefficient = 0.86).

*Social value* was measured with three items (Sweeney and Soutar, 2001): “Following this communicator would make a good impression on other people,” “Following this communicator would help me to feel acceptable,” “Following this communicator would give its owner social approval” (Alpha coefficient = 0.82).

*Altruistic value* was measured with three items (Kim and Chang, 2020): “Gaining science knowledge from this communicator helps me make social contributions,” “Gaining science knowledge from this communicator can provide pure help to other people,” “Gaining science knowledge from this communicator is a kind of social contribution” (Alpha coefficient = 0.80).

*Attitude* was based on three items (Armitage et al., 1999): “It is a good idea to share or to repost science content on social media,” “It makes sense to share or to repost science content on social media,” “It is important to share or to repost science content on social media” (Alpha coefficient = 0.81).

*Internal perceived behavioral control* was based on three items (Armitage and Conner, 1999): “I believe I have the ability to share or to repost science content on social media,” “If it were entirely up to me, I am confident that I would be able to share or to repost science content on social media,” “I am confident to participate in sharing or reposting science content on social media” (Alpha coefficient was 0.85).

*External perceived behavioral control* was based on four items (Armitage and Conner, 1999): “There are likely to be plenty of opportunities for me to share or to repost science content on social media,” “I have many social media channels to share or to repost science content,” “Whether or not I share or repost science content on social media is entirely up to me,” “If I want to, I can share or repost science content on social media”(Alpha coefficient = 0.78).

*Subjective norm* was based on three items (Armitage et al., 1999): “My friends think I should share or repost science content on social media,” “My friends want me to share or to repost science content on social media,” “My friends would approve of my sharing or reposting science content on social media” (Alpha coefficient = 0.83).

*Descriptive norm* was based on two items (Poliakoff and Webb, 2007): “Some friends you know best often share or repost science content on social media,” “Many people around you share or repost science content on social media” (Spearman’s rank correlation coefficient = 0.82).

*Intention* was measured based on Kim and Chang (2020) scale: “I am willing to share and repost the information about this communicator on social media,” “I am willing to share and repost the content produced by this communicator on social media,” “I am willing to share and repost the knowledge learned from this communicator on social media,” “I will share a positive assessment of this communicator on social media with others” (Alpha coefficient = 0.89).

### Analysis

Pearson correlations, means, and standard deviations were presented in Table 2. We conducted hierarchical regression analyses by first including control variables (Model 1). Then we included TCV variables (Model 2), followed by TPB variables (Model 3). The maximum Variance Inflation Factor for each variable was 2.84, which was lower than the standard value 5 proposed by Hair et al. (2011), indicating that the results were not disturbed by multicollinearity (see Table 3).

**Table 2:** Mean, Standard Deviations, and correlations.

**Variables**	**1**	**2**	**3**	**4**	**5**	**6**	**7**	**8**	**9**	**10**	**11**	**12**	**13**	**14**	**15**	**16**	**17**
1. **Age**	—																
2. **Gender**^**a**^	−0.13^**^	—															
3. **Education**^**b**^	0.28^**^	0.02	—														
4. **Income**^**c**^	0.49^**^	−0.15^**^	0.36^**^	—													
5. **Communicator**^**d**^	−0.13^**^	0.07	−0.12^**^	−0.16^**^	—												
6. **Content**^**e**^	−0.21^**^	−0.01	−0.10^*^	−0.12^**^	0.26^**^	—											
7. **Frequency**	0.23^**^	−0.04	0.04	0.22^**^	−0.03	−0.07	—										
8. **Functional value**	0.13^**^	−0.04	0.05	0.13^**^	−0.05	−0.00	0.47^**^	—									
9. **Emotional value**	0.04	−0.08	0.05	0.10^*^	0.04	0.15^**^	0.40^**^	0.65^**^	—								
10. **Social value**	0.24^**^	−0.07	0.03	0.16^**^	−0.21^**^	−0.14^**^	0.27^**^	0.41^**^	0.29^**^	—							
11. **Altruistic value**	0.16^**^	−0.01	0.12^**^	0.19^**^	−0.17^**^	−0.07	0.27^**^	0.47^**^	0.36^**^	0.54^**^	—						
12. **Attitude**	0.06	0.04	0.06	0.05	0.07	0.04	0.19^**^	0.41^**^	0.31^**^	0.35^**^	0.36^**^	—					
13. **Internal perceivedbehavioral control**	0.19^**^	−0.05	0.13^**^	0.20^**^	−0.16^**^	−0.15^**^	0.32^**^	0.32^**^	0.28^**^	0.39^**^	0.37^**^	0.36^**^	—				
14 **External perceivedbehavioral control**	0.13^**^	−0.06	0.09	0.17^**^	−0.06	−0.12^**^	0.32^**^	0.42^**^	0.35^**^	0.36^**^	0.36^**^	0.34^**^	0.68^**^	—			
15 **Subjectivenorm**	0.21^**^	−0.06	0.09^*^	0.21^**^	−0.18^**^	−0.17^**^	0.30^**^	0.29^**^	0.22^**^	0.55^**^	0.44^**^	0.39^**^	0.55^**^	0.49^**^	—		
16 **Descriptivenorm**	0.28^**^	−0.03	0.11^*^	0.28^**^	−0.16^**^	−0.18^**^	0.30^**^	0.31^**^	0.21^**^	0.54^**^	0.47^**^	0.27^**^	0.49^**^	0.44^**^	0.65^**^	—	
17 **Intention**	0.24^**^	0.00	0.12^**^	0.25^**^	−0.11^*^	−0.13^**^	0.33^**^	0.39^**^	0.39^**^	0.43^**^	0.49^**^	0.48^**^	0.58^**^	0.48^**^	0.55^**^	0.50^**^	—
**Mean**	26.29	0.63	2.06	1.88	0.50	0.53	4.61	5.47	5.66	4.36	5.13	5.84	5.16	5.41	4.43	4.25	5.29
**SD**	5.59	0.48	0.43	0.94	0.50	0.50	1.35	0.97	1.00	1.11	1.09	1.00	1.23	1.08	1.18	1.40	1.09

N = 489.

a “0” = male, “1” = female.

b “1” = high school or below, “2” = undergraduate, “3” = postgraduate.

c “1” = less than 5,000, “2” = 5,001–10,000, “3” = 10,001–15,000, “4” = 15,001–20,000, “5” = more than 20001.

d “0” = organization, “1” = individual.

e “0” = article, “1” = video.

* p < 0.05;

** p < 0.01 (two-tailed).

**Table 3:** Regression results.

	Model 1			Model 2			Model 3		
Predictors	*B*	*SE*	*β*	*B*	*SE*	*β*	*B*	*SE*	*β*
**Control variables**									
Age	0.02	0.01	0.10	0.01	0.01	0.07	0.01	0.01	0.07
Gender	0.11	0.10	0.05	0.14	0.09	0.06	0.11	0.07	0.05
Education	0.05	0.12	0.02	0.01	0.10	0.00	0.07	0.09	−0.03
Income	0.14	0.06	0.12^*^	0.11	0.05	0.10^*^	−0.07	0.04	0.06
Communicator	−0.12	0.10	0.05	0.02	0.09	0.01	0.01	0.08	0.00
Content	−0.13	0.10	0.06	−0.20	0.09	−0.09^*^	−0.13	0.08	−0.06
Frequency	0.22	0.04	0.28^**^	0.07	0.04	0.09^*^	0.02	0.03	0.02
**TCV variables**									
Functional value				0.01	0.06	0.01	−0.05	0.05	−0.05
Emotional value				0.23	0.06	0.21**	0.19	0.05	0.18^**^
Social value				0.17	0.05	0.16^**^	−0.03	0.05	−0.03
Altruistic value				0.29	0.05	0.27**	0.16	0.05	0.15^**^
**TPB variables**									
Attitude							0.23	0.04	0.21^**^
Internal perceived behavioral control							0.27	0.05	0.25^**^
External perceived behavioral control							0.01	0.05	0.01
Subjective norm							0.17	0.05	0.15^**^
Descriptive norm							0.10	0.05	0.09
F	13.14^**^			24.51^**^			33.74^**^		
*R* ^2^	0.15			0.35			0.52		

N = 489.

* p < 0.05;

** p < 0.01 (two-tailed).

## Results

Hierarchical regression results indicated that the frequency of watching science content (*Β* = 0.22, *SE* = 0.04, *β* = 0.28, *p* < 0.01) and monthly income (*Β* = 0.14, *SE* = 0.06, *β* = 0.12, *p* < 0.05) were significant predictors of participants’ SSC intentions (Table 3). To be more specific, higher income and more frequent exposure to scientific content promote audience’s SSC intention. However, age, gender, education, type of communicator (organization or individual) and type of content (article or video) were nonsignificant.

Further, the influence of income on SSC intention is significant in Model 1 and 2, but was nonsignificant in Model 3. Therefore, it is possible that the TPB variables mediated the influence of income on SSC intention. In other words, audience with higher income has stronger SSC intention because of more positive attitude, more internal perceived behavioral control, and more subjective norm. And this finding is not difficult to understand. Consider an engineer in a tech company as an example. Since mastering science and technology brings high income, this engineer will naturally have a more positive attitude towards participating in SSC (Attitude); because of the professionalism and scientific literacy, this engineer will have more confidence, adequate planning, and the ability to recommend professional science knowledge to his relatives and friends (Internal perceived behavioral control); and because of the high income, this engineer would feel a greater responsibility to engage in SSC (Subjective norm).

When TCV variables were included in the model, emotional value (*Β* = 0.23, *SE* = 0.06, *β* = 0.21, *p* < 0.01), social value (*Β* = 0.17, *SE* = 0.05, *β* = 0.16, *p* < 0.01), and altruistic value (*Β* = 0.29, *SE* = 0.05, *β* = 0.27, *p* < 0.01) were significant predictors of SSC intentions, while functional value was nonsignificant. These results indicated that science content with higher emotional, social, and altruistic value made participants more willing to share or to repost science information. Thus, the results support Hypothesis 1b, 1c, and 1d.

With TPB variables included in the model, attitude (*Β* = 0.23, *SE* = 0.04, *β* = 0.21, *p* < 0.01), internal perceived behavioral control (*Β* = 0.27, *SE* = 0.05, *β* = 0.25, *p* < 0.01), and subjective norm (*Β* = 0.17, *SE* = 0.05, *β* = 0.15, *p* < 0.01) were significant predictors of SSC intentions, but external perceived behavioral control and descriptive norm were not. In addition, social value was no longer significant in this model (*Β* = −0.03, *SE* = 0.05, *β* = −0.03, *p* = 0.50). Thus, Hypotheses 2a, 2b and 2d were supported while Hypothesis 2c and 2e were rejected.

## Discussion

Digitalization and the rise of social media platforms have revolutionized the way in which scientists interact with diverse non-scientific publics (Bubela et al., 2009; Rutsaert et al., 2013). A wide range of social media platforms give scientists new means to share scientific insights with citizens directly, but also allow audiences to generate information themselves (Bubela et al., 2009; Rutsaert et al., 2013; Hara et al., 2019). This has extended the range of actors involved in the production and use of scientific knowledge to artists, activists, bloggers, amateur enthusiasts and social media influencers — social media extends the role of audiences, typically considering them roles as both the “audience” and the “communicator.”

This study has important implications for the practices of online science communication. We suggested that a positive SSC can effectively address the problems scientists face when disseminating science knowledge (think about communication in a simple way, fear of criticism and personal attacks, narrow set of skills and considerations), but a negative SSC can lead to more serious outcomes (spreading and reinforcement of false information). Therefore, understanding of the predictors of SSC can help communicators to better manage SSC. We contend that scientists can provide different values for different science content and adjust their strategies in time according to the audiences’ feedback to increase their interest in participating in SSC.

On this background, we first proposed the concept of SSC and then encouraged audiences to actively participate in SSC. Different from the concept of “retransmission” which is only applicable to the simple repetition of original information (Kim et al., 2013; Luarn et al., 2014; Sutton et al., 2015), “SSC” emphasizes audience’s value-based evaluation and selective dissemination, and considers audience’s information reprocessing behavior. Furthermore, the “retransmission” concept reflects the traditional unidirectional, sender-receiver mode of science communication. In contrast, SSC represents a bidirectional, participatory, deliberative communication in which the audiences are active participants (Kurath and Gisler, 2009). And we suggest that the “SSC” concept is more suitable in a social media context, because it builds a bridge between science communicators and lay audiences in equal conversations (Scheufele, 2014).

Previous researches on scientific communication highlights the importance of encouraging audiences’ participation. However, literatures are largely silent on the way to accomplish it on social media or factors leading to audiences’ online SSC behavior as a main form of science participation. For example, reposting and modified reposting on social media lead to audiences’ greater perceived source credibility, perceived content effectiveness, and likelihood to engage in the science content (Dong, 2015; Dockter et al., 2020). To fill in this gap, the current research employs the TCV and TPB to explain audiences’ SSC behaviors, with its results indicating that both TCV-based and TPB-based factors play an important role in predicting audiences’ SSC intention on social media.

Specifically, it is found that science content with higher emotional, social, and altruistic value leads to audiences’ stronger SSC intentions. This suggests that communicators can promote audience SSC behavior by enhancing the color of science content and emphasizing the social and altruistic value of learning science knowledge. Moreover, functional value has little effect on SSC intentions, which, to a degree, can explain why even if some scientific contents seem helpful, they can only be effectively spread in a single-connected community (Williams et al., 2015). In other words, audiences need far more than science content that only meets their basic demand for knowledge acquiring. More efforts should be put in by communicators to make science content more colorful and make it easier for those audiences who give attentions to science to feel its perceived social value and its possible contribution to the whole society. Only in this way, can it be possible to get more audiences involved into SSC.

Although previous research has examined audience attitude towards online science content, they focused more on non-value predictors, such as gender of the science communicator (Chau, 2010; Abisheva et al., 2014), veracity of the information (Pandey et al., 2010; Murugiah et al., 2011; Azer, 2012), type of channel (Kim, 2012; Welbourne and Grant, 2016), topic and sentiment of a tweet (Naveed et al., 2011), etc. However, as science content gains popularity among a broader audience, the scientific communication must be useful and appealing. Therefore, studying the value predictors is vital to understand what drives popularity broadly (Welbourne and Grant, 2016). Therefore, we focus on TCV-based predictors, as opposed to non-value factors because they are very helpful for communicators to produce high-quality science content and also useful for understanding drivers of wide spread online.

Moroeover, attitude, internal perceived behavioral control, and subjective norm can lead to positive SSC intention. These findings are helpful for communicators to improve the communication effectiveness of their science-related content, as audience engagement in science is low due to disinterest and lack of motivation to process science information (Bauer, 2016; Hargittai et al., 2018). On the one hand, we suggest that communicators should encourage audiences to participate in science discussion as well as to repost science content on their social media, in order to promote consumer to form a positive attitude towards SAC and subjective norm. On the other hand, government and online communicators should work together to build a suitable environment for audiences to disseminate SSC. For example, adequate freedom should be provided for scientific discussion and equal attention should be given to comments and reposting on social media.

As with all studies, this research has several limitations that may be addressed by future research. First, as a result of cognitive dissonance, audience might increase the perceived value of science content after they have participated in SSC. Further investigation conducted through lab experiment designs can verify the results of the current research. Second, the participants were followers of science communicator on social media. And if the participants had a degree of interest might lead to “Ceiling Effect,” this could have some impact on findings associated to SSC attitudes. Additionally, the science communication on social media is more fragmented and the online users already have a high willingness to share (Rutsaert et al., 2013; Mingoia et al., 2017), thus the survey focused only on social media may ignore some other information. And there may be some differences in communication behaviors between different social medias. Thus, we call for further research on SSC on social media, especially in cross-media contexts. The survey is based on Chinese samples and a high percentage of the sample are highly educated. Therefore, it should be further tested whether the same effects still exist in other countries or in low educational level audiences in the future.

Despite these limitations, this study points out the necessity of SSC behavior on social media and provides clear evidence that TCV and TPB are useful for predicting audiences’ SSC intention. Results are informative considering it is one of the first few studies that deal with audience dissemination issues, which, if managed efficiently, can contribute to a wider audience engagement with science thus promoting a higher level of science communication environment.

## Data availability statement

The raw data supporting the conclusions of this article will be made available by the authors, without undue reservation.

## Ethics statement

Ethical review and approval were not required for the study on human participants in accordance with the local legislation and institutional requirements. Written informed consent for participation was not required for this study in accordance with the national legislation and the institutional requirements.

## Author contributions

ZH and BM conceived the study and wrote the first draft of the article. RB was responsible for revising and proofreading the manuscript. All authors contributed to the article and approved the submitted version.

## Conflict of interest

The authors declare that the research was conducted in the absence of any commercial or financial relationships that could be construed as a potential conflict of interest.

## Publisher’s note

All claims expressed in this article are solely those of the authors and do not necessarily represent those of their affiliated organizations, or those of the publisher, the editors and the reviewers. Any product that may be evaluated in this article, or claim that may be made by its manufacturer, is not guaranteed or endorsed by the publisher.

## Footnotes

^1^ Sojump.com

^2^ Huxiu.com
